# Analysis of a transgenic *Oct4* enhancer reveals high fidelity long-range chromosomal interactions

**DOI:** 10.1038/srep14558

**Published:** 2015-10-05

**Authors:** Mingyang Cai, Fan Gao, Peilin Zhang, Woojin An, Jiandang Shi, Kai Wang, Wange Lu

**Affiliations:** 1 Eli and Edythe Broad Center for Regenerative Medicine and Stem Cell Research, University of Southern California, Los Angeles, CA 90033, USA; 2 Zilkha Neurogenetic Institute, University of Southern California, Los Angeles, CA 90033, USA; 3 Division of Biostatistics, Department of Preventive Medicine, University of Southern California, Los Angeles, CA 90033, USA; 4 Department of Biochemistry and Molecular Biology, Norris Cancer Center, Keck School of Medicine, University of Southern California, Los Angeles, CA 90033, USA; 5 College of Life Sciences, Nankai University, Tianjin 300071, China; 6 Department of Psychiatry, Keck School of Medicine, University of Southern California, Los Angeles, CA 90033, USA; 7 Department of Stem Cell Biology and Regenerative Medicine, Keck School of Medicine, University of Southern California, Los Angeles, CA 90033, USA

## Abstract

Genome structure or nuclear organization has fascinated researchers investigating genome function. Recently, much effort has gone into defining relationships between specific genome structures and gene expression in pluripotent cells. We previously analyzed chromosomal interactions of the endogenous *Oct4* distal enhancer in pluripotent cells. Here, we derive ES and iPS cells from a transgenic *Oct4* distal enhancer reporter mouse. Using sonication-based Circularized Chromosome Conformation Capture (4C) coupled with next generation sequencing, we determined and compared the genome-wide interactome of the endogenous and transgenic *Oct4* distal enhancers. Integrative genomic analysis indicated that the transgenic enhancer binds to a similar set of loci and shares similar key enrichment profiles with its endogenous counterpart. Both the endogenous and transgenic *Oct4* enhancer interacting loci were enriched in the open nucleus compartment, which is associated with active histone marks (H3K4me1, H3K27ac, H3K4me3 and H3K9ac), active *cis*-regulatory sequences (DNA hypersensitivity sites (DHS)), 5-hydroxymethylcytosine (5-hmc), and early DNA replication domains. In addition, binding of some pluripotency-related transcription factors was consistently enriched in our 4C sites, and genes in those sites were generally more highly expressed. Overall, our work reveals critical features that may function in gene expression regulation in mouse pluripotent cells.

Pluripotent embryonic stem (ES) cells can self-renew indefinitely and generate all cell lineages in an organism, including the germ line^1^,^2^,^3^. Induced pluripotent stem (iPS) cells are genetically reprogrammed to an ES cell-like state by over-expression of genes that maintain ES cell properties^4^. Both types of cells are valuable tools in disease modeling, drug development and, particularly, cell replacement therapy. Understanding molecular mechanisms underlying pluripotency is a pressing task if we are to take full advantage of these cell types.

Recent studies have provided insight into chromatin folding patterns in 3D space as a mechanism governing stem cell pluripotency^5^. In fact, in addition to the linear arrangement of information encoded on DNA fibers, the 3-D architecture of the genome is increasingly thought to be a defining factor in gene regulation^6^. Both critical stem cell activities, self-renewal and pluripotency, require a distinct chromosome organization: one “stable” enough to maintain cellular memory but “plastic” enough to assume a germ-layer specific conformation^7^.

A determinant of genome architecture is long-range chromatin-chromatin interactions occurring genome-wide. Since 2002, when chromosome conformation capture (3C) was first used to investigate interactions between two genomic segments^8^, a repertoire of high-throughput assays (4C^9^, 4C-seq^10^,^11^, 5C^12^, Hi-C^13^, and ChIA-PET^14^) has been developed to create chromatin interaction maps. These methods have greatly advanced our understanding of genome organization. Among them, circularized chromosome conformation capture coupled with next-generation sequencing (4C-seq) is an unbiased “one-versus-all” approach used to detect all genomic regions interacting with a region of interest^11^,^15^. Previously, we applied enzyme-based 4C-seq to capture genome-wide regions targeting the *Oct4* locus, a region regulating ES cell pluripotency^5^,^16^. Furthermore, we have applied sonication-based 4C-seq to identify regions strongly interacting with a specified “bait”^16^,^17^. This modified approach was found as effective as enzyme-based methods but minimized bias due to enzyme digestion^17^.

*Oct4* gene expression is governed by upstream distal and proximal enhancers^18^. The former is responsible for *Oct4* expression in ES cells, while the latter controls expression in epiblasts^18^. *Oct4* regulation by the distal enhancer is achieved by spatial proximity via looping^7^,^19^. Furthermore, there is evidence that enhancer elements can regulate genes hundreds to thousands of base pairs away. Therefore, we reasoned that *Oct4* distal enhancer could regulate both nearby and distant genes on the same or even different chromosomes and that these interactions could be effectively detected by 4C-seq technology using the *Oct4* distal enhancer as “bait”.

Here, we report that the *Oct4* distal enhancer interacts with genomic loci that exhibit open chromosome features and contain active histone marks. Genes residing at these loci were expressed at levels higher than genes in other regions. We also demonstrate that long-range chromosomal interaction correlates with *Oct4* gene transcription and show that somatic cells reprogrammed to iPS cells establish long-range chromosome interactions at the *Oct4* locus before activating *Oct4* transcription. When we compared the interactome of a transgenic *Oct4* distal enhancer with its endogenous counterpart in iPS cells using sonication-based 4C-seq, we observed similar interacting loci. Overall, this analysis yields insight into high-fidelity interacting regions likely critical for gene expression in mouse pluripotent stem cells.

## Results

### Identification of endogenous and transgenic *Oct4* enhancer interactomes in mouse ES and iPS cells

**Figure 1:**
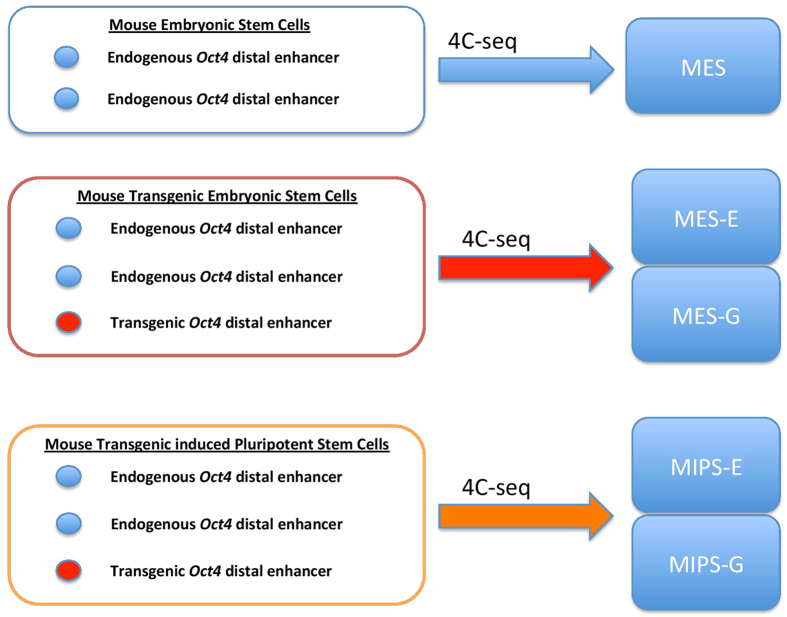
Study design. Left boxes indicate cell lines used; right boxes indicate 4C-seq datasets obtained from indicated mouse ES or iPS cells. Notations are defined as 4C-seq targeting: MES, endogenous *Oct4* distal enhancer in wild-type ES cells; MES-E, endogenous enhancer in transgenic ES cells; MES-G, transgenic enhancer in transgenic ES cells; MIPS-E, endogenous enhancer in transgenic iPS cells; and MIPS-G, transgenic enhancer in transgenic mouse iPS cells. Datasets targeting transgenic *Oct4* distal enhancer are designated “G” for GFP, while “E” indicates endogenous enhancer in a transgenic line. The transgenic Oct4 enhancers are shown as red dots while endogenous enhancers are shown as blue dots in left boxes.

We applied a sonication-based 4C-seq technique to identify interacting partners of an *Oct4* distal enhancer “bait” in three pluripotent lines, including mouse ES cells, mouse transgenic ES cells and mouse transgenic iPS cells (Fig. 1). The transgenic ES cell line contained both endogenous and transgenic *Oct4* distal enhancers^18^, enabling us to obtain two sets of 4C interactomes in that line. Thus, we obtained five sets of 4C interactomes: one for mouse ES cells, two for mouse transgenic ES cells, and two for mouse transgenic iPS cells. Notations used to identify *Oct4* enhancer mouse lines were: MES, endogenous enhancer in wild-type ES cells; MES-E, endogenous enhancer in transgenic ES cells; MES-G, transgenic enhancer in transgenic ES cells; MIPS-E, endogenous enhancer in transgenic iPS cells; and MIPS-G, transgenic enhancer in transgenic mouse iPS cells. We obtained two biological replicates per experiment.

Details relevant to sonication-based 4C are found in Gao *et al.*^17^. Briefly, chromosome crosslinking via fixation captured spatial proximities within the nucleus, and a circular structure was formed by sonication followed by ligation. We then employed “bait”-specific primers to capture genomic regions interacting with an *Oct4* distal enhancer “bait” by inverse nested PCR. Two sets of primers were designed to target endogenous or exogenous enhancers (see **Methods**). 4C libraries were then constructed and subjected to next-generation sequencing.

**Figure 2:**
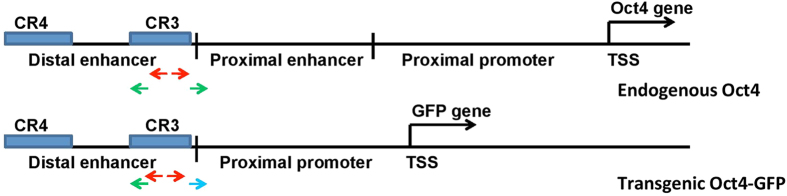
Nested PCR primers targeting the *Oct4* distal enhancer. *Oct4-*specific primers (red arrowheads for the 1^st^ set, green arrowheads for the 2^nd^ set) target the endogenous enhancer in mouse ES, iPS, transgenic ES, and transgenic iPS cells. GFP-specific primers (red arrowheads for the 1^st^ set, green and cyan arrowheads for the 2^nd^ set.) target the transgenic enhancer in both transgenic cell types. The only difference in primers targeting endogenous and transgenic enhancers is the forward primer of 2^nd^ set, shown as cyan arrowhead for transgenic experiment while green arrowhead for endogenous experiment. Blue boxes represent conserved regions CR3 (right) and CR4 (left) found in the distal enhancer in human, bovine, rat and mouse *Oct4* orthologs. TSS, transcription start site.

Based on our previously established protocol, we employed a paired-end tag (PET) mapping strategy^15^,^17^ in which short paired tags are extracted from DNA fragment ends. In our hands, this is an optimal approach to identify bait-interacting regions by spotting reads that are mosaics of the bait and interacting regions^17^. Here, we define the “bait” as a ~0.6 kb region including a 300-bp extension from locations of the second set of PCR primers (Fig. 2). Overall, we identified thousands of distal sites in 10 datasets (see Table 1). Here, we focused primarily on inter-chromosomal interactions as they account for most of the interaction pool.

### Reproducibility of inter-chromosomal interactions

**Figure 3:**
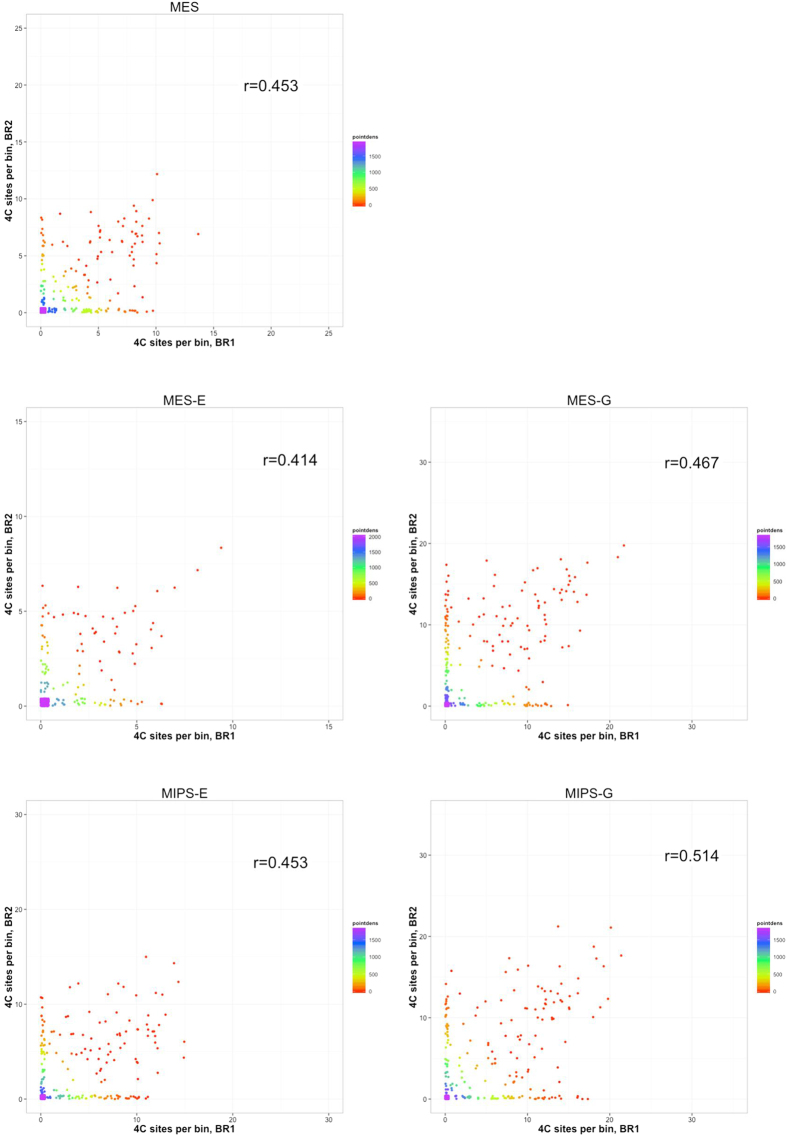
Scatter plot of density of inter-chromosomal interactions. For each of five experiment contexts, data points are generally scattered along a 45° diagonal. Color scale indicates data point density. The Pearson correlation coefficient is shown in each panel.

We initially determined reproducibility of inter-chromosomal interactions between biological replicates by counting interactions in every 2Mb genomic bin and correlating them between biological replicates. For inter-chromosomal interactions generated in replicates, Pearson’s correlation coefficient was >0.4 in all five 4C experiment groups (Fig. 3). This finding suggests that the chromosome conformation capture strategy captures only a subset of diverse interactions occurring within the nucleus.

**Figure 4:**
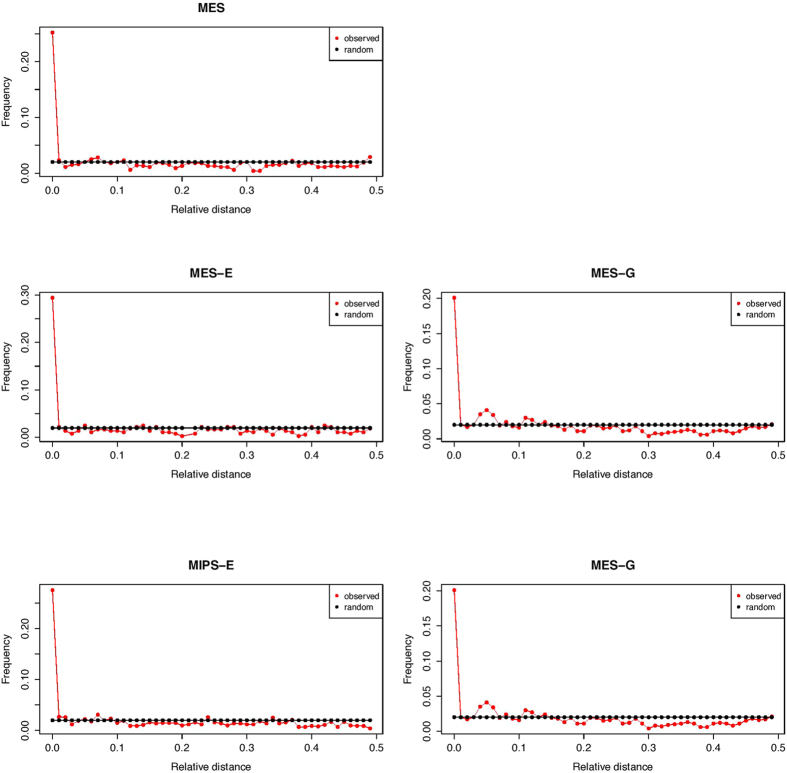
Distribution of relative distances between 4C sites in two biological replicates. observed: distribution of relative distance between 4C sites in two biological replicates of 4C samples; random: distribution of relative distance between two set of sites with no spatial correlation.

To evaluate consistency between biological replicates in a different manner, we employed a strategy devised by Favorov *et al.*^20^, namely, to measure relative distance between two sets of genomic intervals. When we plotted distribution of relative distance between 4C sites in two biological replicates, we observed a significant enrichment in frequency around the relative distance 0 (Fig. 4), indicating consistency between replicates.

### Identification of significantly enriched interacting regions

Application of our custom computational pipeline identified thousands of sites interacting with the bait region (Table 1). To minimize noise due to random collisions within the nucleus and identify only biologically relevant sites, we applied a statistical model with a permutation-based false discovery rate (FDR) procedure. A z-score was assigned to each site based on the number of interacting sites within a 2Mb window around that site. FDR was calculated by random permutation of the data 100 times and the 0.05 threshold was chosen to select positive sites. Positive sites and nearby interacting sites within +/−1 Mb (Supplementary Table 1) were grouped as enriched interacting regions, and overlapping regions were further merged (Table 1).

### Comparison of enriched interacting regions identified from 4C-seq data

**Figure 5:**
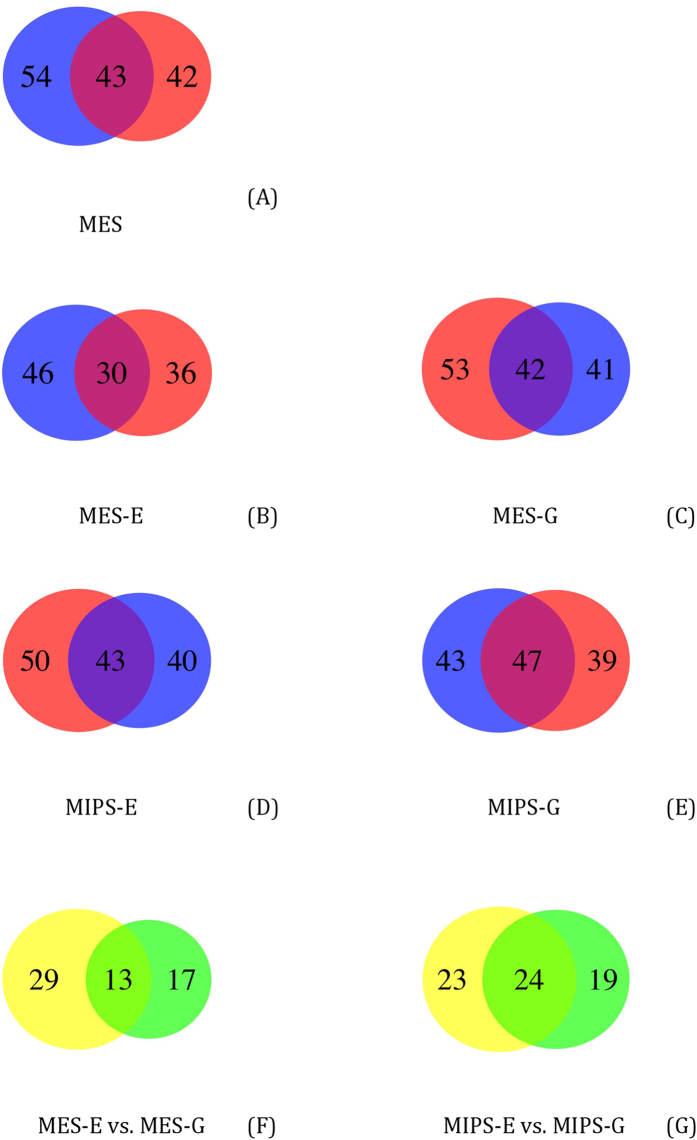
Venn diagram of interacting regions between biological replicates and between endogenous and exogenous interactomes. The number of regions overlapping between biological replicates in all five experiments (**A–E**) is shown. 13 high-fidelity regions are identified between MES-E and MES-G and 24 between MIPS-E and MIPS-G (**F,G**).

Statistical analysis identified dozens of enriched interacting regions in each of the five experimental contexts (Fig. 5, Supplementary Table 2), with numerous regions shared between two replicates. Such regions represent high-confidence interactions of potential biological significance. Of note, we identified some regions that interact with both the endogenous and transgenic *Oct4* distal enhancer (13 for MES and 24 for MIPS, Supplementary Table 2), suggesting that both bind a similar set of genomic loci and that these regions could be relevant to stem cell fate. Of note, 9 regions (Supplementary Table 2) are consistently identified in MES and MIPS. In addition, we concluded that information in individual datasets could be masked if we focused only on high-fidelity regions. Thus, in the following association analysis, we compared interacting sites in individual datasets.

### Transgenic and endogenous *Oct4* enhancer interactomes are similarly enriched with active nuclear compartments

**Figure 6:**
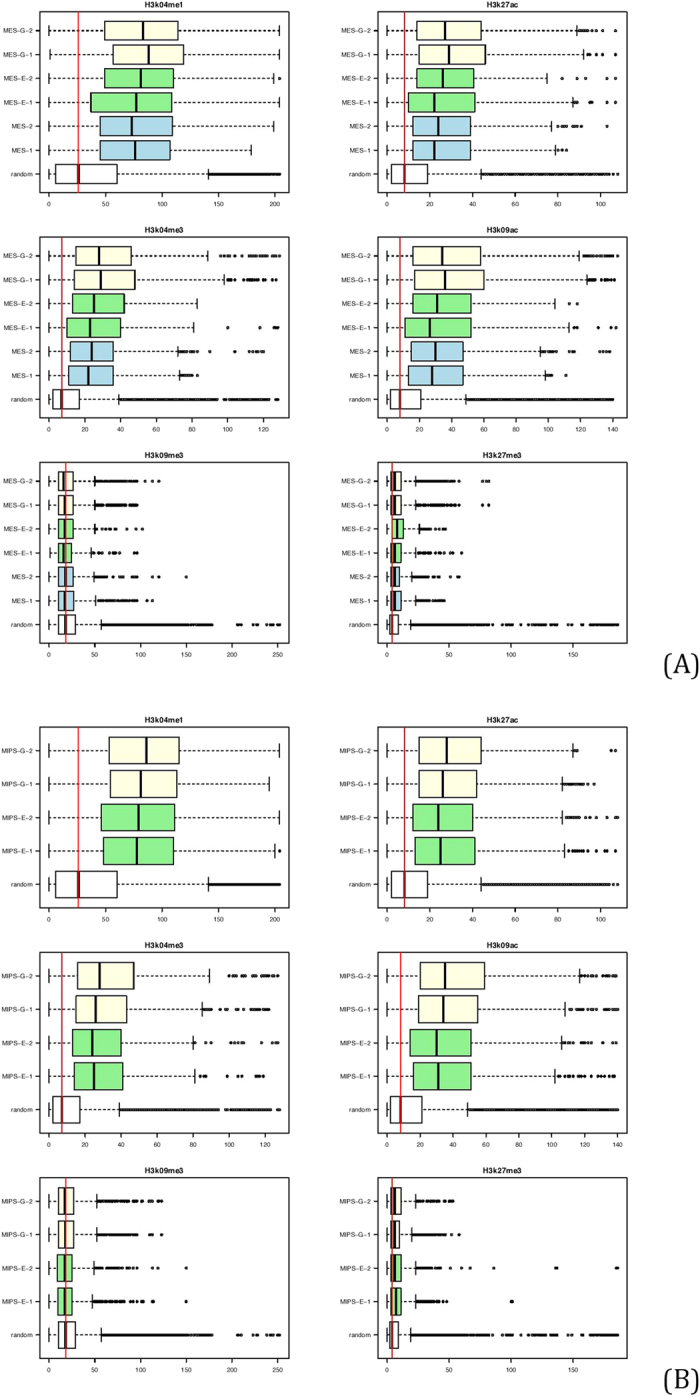
Enrichment analysis of histone marks around 4C sites in mouse ES (A) and iPS (B) cells. 4C sites are enriched with enhancer (H3K4me1 and H3K27ac) and promoter (H3K4me3 and H3K9ac) marks in both endogenous and transgenic enhancer interactomes relative to random sites. In contrast, no obvious enrichment of repressive marks (H3K9me3 and H3K27me3) is observed in sites interacting with the endogenous or transgenic enhancer.

Hi-C studies suggest a “two-compartment” model of nuclear chromatin, in which “open” compartments are enriched with active histone features, while “closed” compartments lack such marks^13^,^21^. To examine association of our 4C interactomes of the *Oct4* enhancer with histone marks, we examined enrichment of those marks around 4C sites. For all 10 datasets, histone marks related to gene activation, including H3K4me1, H3K27ac, H3K4me3 and H3K9ac, were enriched around identified 4C sites (+/− 0.5 Mb) compared with randomly shuffled genomic sites. By contrast, enrichment for the repressive mark H3K27me3 and the heterochromatin mark H3K9me3 was not significant (Fig. 6A for mouse ES cells; Fig. 6B for mouse iPS cells). This result supports the idea that the *Oct4* enhancer physically interacts with active genomic regions and reveals a consistent pattern of histone marks in endogenous and transgenic enhancer interactomes.

**Figure 7:**
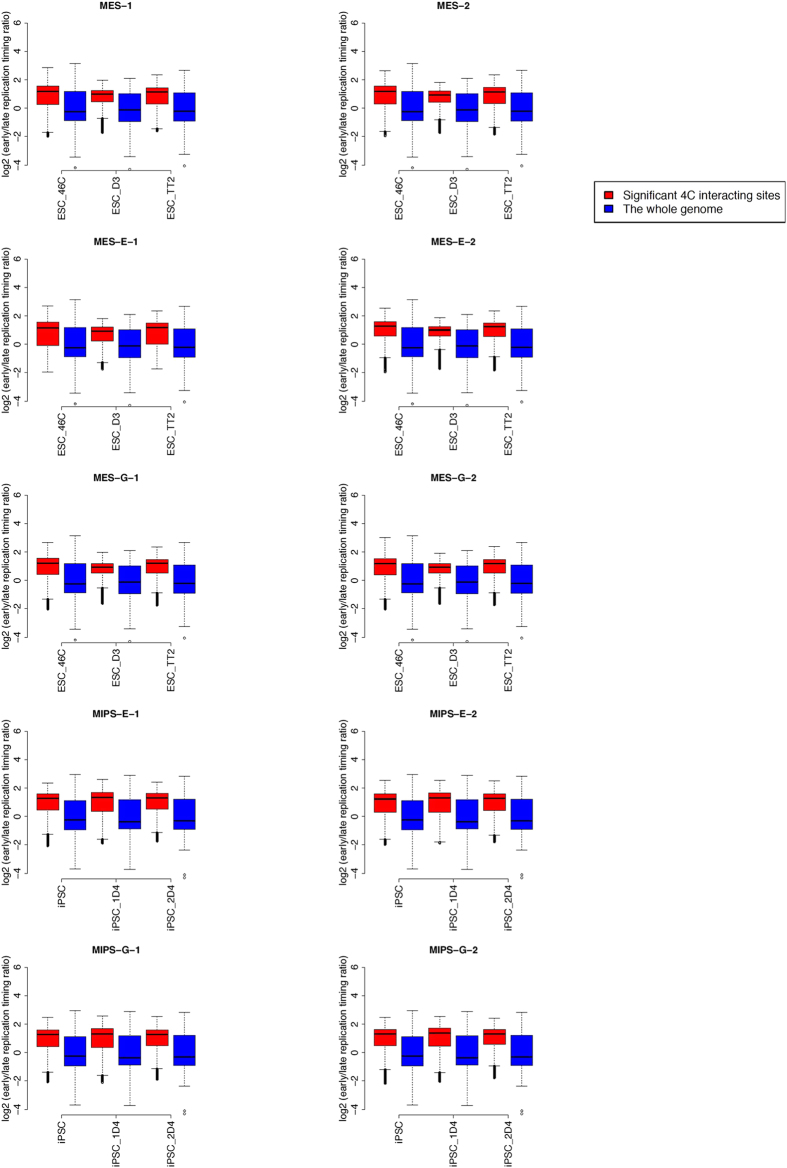
Enrichment analysis of early DNA replication timing domains around 4C sites. Distribution of log2 transformed early/late replication timing ratio of regions +/− 50 kb from interacting sites indicates a shift toward positive values relative to genome-wide counterparts. DNA replication timing data for ES cells (ESC_46C, ESC_D3, and ESC_TT2) were used for analysis of MES, MES-E and MES-G, while data for iPS cells (iPSC, iPSC_1D4, and iPSC_2D4) were used for analysis of MIPS-E and MIPS-G.

Chromatin compartments are also defined by DNA replication domains^22^, and some studies show that DNA replication timing shapes the genomic landscape in specific cell types^23^,^24^,^25^. These findings suggest that early and late DNA replication might occur in different chromatin compartments. Using DNA replication timing data of three ES cell lines and three iPS cell lines (see **Methods**), we established that *Oct4* is located at an early DNA replication domain (log2 transformed early/late replication timing ratio: mean = 1.334, standard deviation = 0.382, p-value = 0.00018). Thus, we asked whether the interactomes identified were similarly correlated with replication timing. We found that *Oct4* enhancer interacting sites overlapped primarily with early DNA replication domains in mouse ES and iPS cells (p < 2.2e-16, Wilcoxon rank-sum test, Fig. 7). Early DNA replication domains are reportedly correlated with active gene transcription^22^, suggesting that contact of the *Oct4* enhancer with distant interacting regions has functional significance.

**Figure 8:**
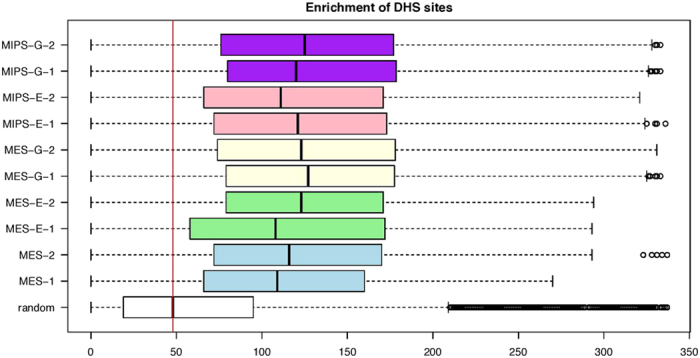
DNA hypersensitivity site counts in the proximity of 4C sites. Boxplot shows the distribution of counts of DHSs around +/− 0.5 Mb of 4C sites. Biological replicates of comparable experiment contexts are labeled with the same color. The red line shows median level of enrichment in random sample.

DNase I hypersensitivity (DHS) sites are universal features of active *cis*-regulatory sequences^26^. We counted DHS sites around +/− 0.5 Mb of 4C sites and at random sites in 10 pluripotent datasets (Fig. 8) and observed significant enrichment around 4C compared with random sites (p < 2.2e-16, Wilcoxon rank-sum test). The enrichment pattern of transgenic enhancer datasets was similar to that of endogenous enhancer datasets, further supporting the idea that the *Oct4* distal enhancer resides in accessible chromatin and likely contains elements regulating stem cell activity.

**Figure 9:**
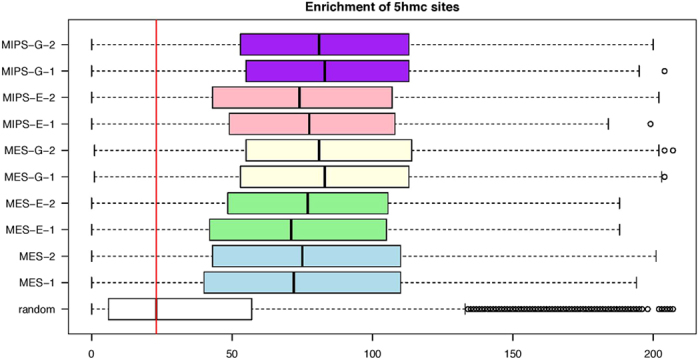
5-hmC site enrichment in the proximity 4C sites. Boxplot shows the distribution of counts of 5-hmC sites around +/− 0.5 Mb of 4C sites. Biological replicates of comparable experiment contexts are labeled with the same color. The red line shows median level of enrichment in random sample.

DNA methylation marked by hydroxymethylcytosine (5-hmC) also regulates pluripotent cell activity^27^. Thus, we counted 5-hmC peaks^28^ genome-wide within +/− 0.5 Mb of 4C sites (Fig. 9). 5-hmC sites were relatively more enriched in the proximity of *Oct4* enhancer interactomes than were randomly selected sites (p < 2.2e-16, Wilcoxon rank-sum test), and that pattern was comparable between endogenous and transgenic enhancer interactomes.

### *Oct4* enhancer interactomes are adjacent to transcription start sites and CpG sites

**Figure 10:**
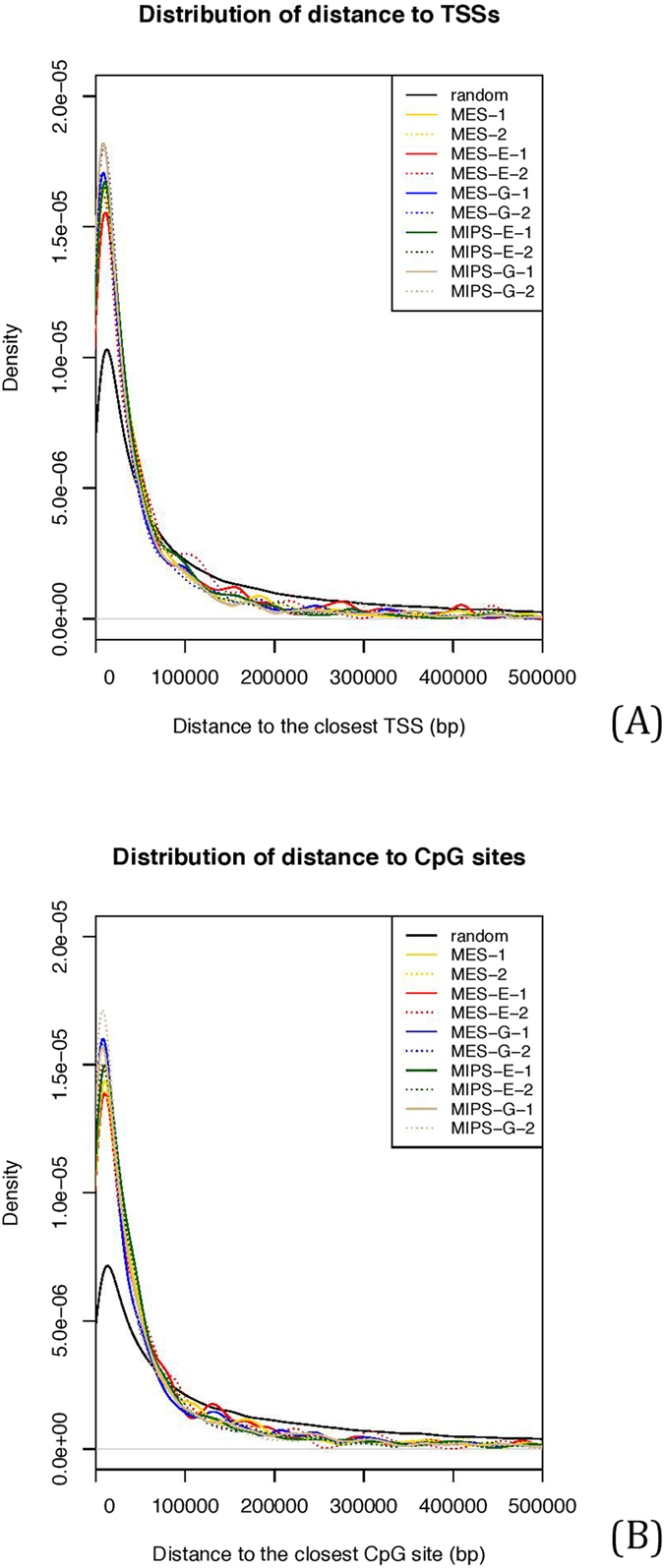
Distribution of distances to TSSs (A)and CpG sites (B). In both kernel density plots, the peak of random sites is significantly less steep around zero location than that of 4C sites in all 10 datasets (p < 2.2e-16).

To correlate *Oct4* enhancer interactomes with annotated gene locations, we examined distribution of genomic distances of 4C sites to nearby genomic elements, including to transcription start sites (TSSs) and CpG sites, which regulate gene expression and govern chromatin organization^29^,^30^. Kernel density was plotted to show the distribution of distance from our 4C sites to TSSs (Fig. 10A) and to CpG sites (Fig. 10B). As shown, the plots show sharp peaks around the zero relative position. Compared to the randomly simulated sites in the whole genome, we observed that peaks of enhancer interactomes were steeper in a statistically significant manner (p < 2.2e-16), suggesting that endogenous and transgenic *Oct4* enhancers preferentially interact with gene-rich regions. Proximity of interactomes to defined TSSs supports the concept of “transcription factories”, in which multiple active gene loci are co-regulated^31^,^32^. Proximity of interactomes to CpG sites also supports the two-compartment model of interphase chromosome organization.

### Expression of *Oct4* enhancer interacting genes is higher than random genes

**Figure 11:**
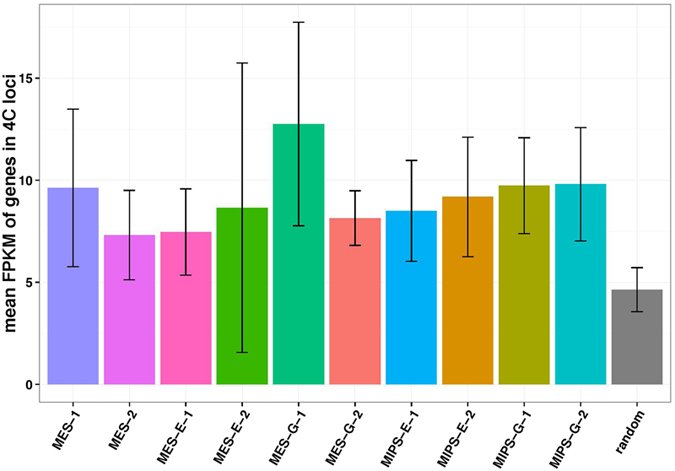
RNA-seq association study of targeted gene expression levels. The average FPKM (fragments per kilobase of exon per million fragments mapped) value was calculated for each of 10 datasets and compared with that of random set of genes. Gene expression levels are elevated in 4C regions relative to randomly selected regions in indicated cells. (p < 0.05 except for MES-E-2; Welch’s two sample *t*-test).

Since *Oct4* enhancer interactomes are closely associated with genes, we identified all genes overlapping with or closest to 4C sites. Gene counts from 10 datasets are summarized in Table 2. We also collected RNA-seq data to examine expression levels of those genes. All interactomes identified in pluripotent cells were associated with genes expressed at a higher level than genes near randomly selected sites (Fig. 11). Genes consistently identified between biological replicates are listed in Supplementary Table 3. The overlapping genes found in endogenous and transgenic enhancer interactomes in MES and MIPS are listed in Supplementary Table 4.

### The *Oct4* enhancer interactome is enriched in transcription factor binding sites

**Figure 12:**
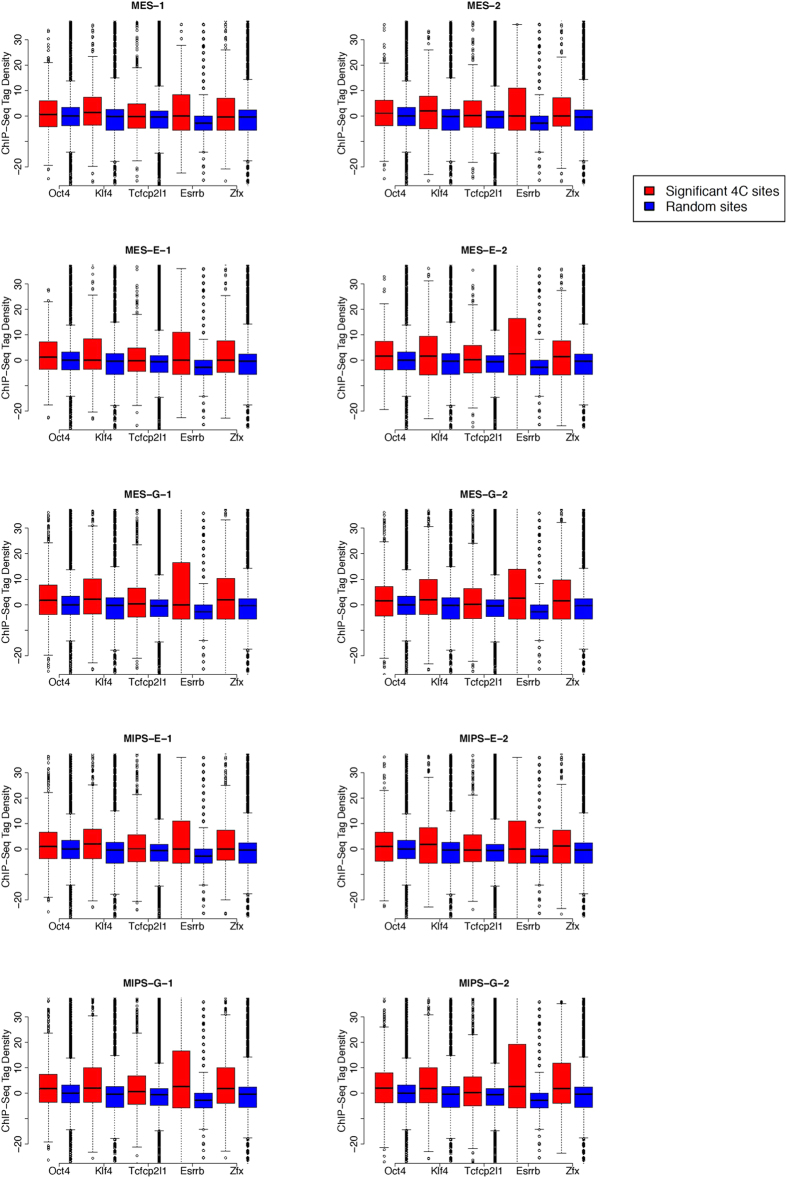
Boxplots of five transcription factors (Oct4, Klf4, Tcfcp2I1, Esrrb, Zfx) enrichment around the enhancer interacting sites (red) and random sites (blue) in 10 datasets. ChIP-seq tags within +/− 1 kb of an interacting site were counted and normalized to 10 million total tags.

Transcription factors can mediate chromatin-chromatin interactions by binding to two DNA segments^33^. We thus asked whether the *Oct4* enhancer interactome is enriched with transcription factor binding sites. To determine this, we analyzed ChIP-seq profiles of 13 sequence-specific transcription factors (Nanog, Oct4, STAT3, Smad1, Sox2, Zfx, c-Myc, n-Myc, Klf4, Esrrb, Tcfcp2l1, E2f1, and CTCF) and 2 transcription regulators (p300 and Suz12) reported previously^34^. We obtained the binding sites for the 15 TFs (Methods), and compared normalized and background-subtracted ChIP-seq tag counts around 4C and random sites using the Wilcoxon rank-sum test. Binding sites for Oct4, Tcfcp2l1, Klf4 and Esrrb were enriched in a statistically significant manner around +/− 1 kb of 4C sites in all 10 datasets (p < 1e-04), while Zfx was significantly enriched in 9 datasets (p < 1e-04) (Fig. 12, Table 3). These transcription factors may mediate chromatin interactions governing stem cell fate.

## Discussion

To study the role of the *Oct4* gene locus in establishing and maintaining pluripotency, previous studies have mapped its interaction partners in mouse^5^ and human^16^ ES cells. To our knowledge, ours is the first interactome map established for a transgenic enhancer, allowing comparison with endogenous enhancer interactomes. Our work shows that even if the *Oct4* distal enhancer resides at a different locus, its interaction partners are comparable to those of the endogenous enhancer.

Here, we applied sonication-based 4C-seq to identify loci interacting with the *Oct4* distal enhancer bait in transgenic mouse ES cells. We found that interacting partners tend to occupy active genomic regions labeled by active histone modifications and other epigenetic marks. This observation suggests that physical contact between the *Oct4* enhancer and other loci may be crucial for activity of transcription of factors regulating pluripotency. Particularly, the *Oct4* enhancer are closer to other enhancer regions in 3D space, thus may interact and synergize with enhancers of other genes functioning in pluripotent stem cells. In fact, synergy among enhancers has been previously shown in analysis of the mouse immunoglobulin kappa (*Igκ*) gene^35^,^36^,^37^,^38^. Besides, the fact that *Oct4* enhancer interacts with a set of actively transcribed genes in pluripotent cells supports the association of genomic architecture with gene expression, as reported in a previous study^39^. Genes residing in the 4C regions identified were actively expressed, revealing how far-reaching chromosome-chromosome interactions can have functional consequences in cells.

We observed high reproducibility in our study, both between biological replicates and between endogenous and transgenic enhancer interactomes. However, we saw some variation in outcomes potentially due to the methodologies employed. The Pearson’s correlation coefficient of >0.4 is large enough in evaluating reproducibility between replicates, and is comparable to similar studies of this type. Chromatin-chromatin interactions are highly dynamic and transient^40^, and 4C-seq takes a snapshot of chromatin interaction patterns across millions of cells. By comparison, a previous study of the CTCF-mediated chromatin interactome in pluripotent cells using ChIA-PET reported that overlap between two biological replicates was 38%^41^.

4C-seq provides genome-wide candidate of regions and genes that interact with *Oct4* enhancer. However, validation of the function of individual locus and gene is still required for unveiling the specific function. DNA fluorescence *in situ* hybridization (FISH) is a powerful approach to reveal nuclear positioning of a pair of loci^42^,^43^,^44^, and thus could be applied to validate specific interacting candidates suggested by 4C-seq. In addition, chromosome conformation capture (3C)^8^ could be used to quantify contact frequencies between selected genomic sites in a more targeted manner.

In summary, we report consistent interactome profiles for both the endogenous and transgenic *Oct* enhancer and conclude that interactions we observed are likely relevant to gene expression and pluripotency. It is important to keep in mind that we are relying on millions of cells and that our observations reflect behavior of cell populations. Such profiles should be interpreted with caution when predicting the function of a single cell.

## Methods

### Cell culture

Mouse E14 ES cells were grown in culture dishes coated with 0.1% gelatin in Glasgow Minimum Essential Medium (GMEM) supplemented with 15% fetal bovine serum (FBS), 100 nM nonessential amino acids, 1% sodium pyruvate, 200 mM glutamate, 1% penicillin streptomycin, 50 uM β-mercaptoethanol and 10 ng/mL LIF. Medium was replaced every 24 hours.

### Generation of sonication-based 4C libraries

Immediately before library preparation, 10 million cells were cross-linked in culture dishes with freshly prepared 1% formaldehyde. To extract chromatin, cells were detached and treated with Triton X100 buffer (0.25% Triton X100, 10 mM EDTA, 10 mM Tris-HCl, pH8.0, 100 mM NaCl, 1 × protease inhibitor cocktail). Isolated chromatin pellets were resuspended in SDS lysis buffer (1% SDS, 5 mM EDTA, 50 mM Tris-HCl, pH8.0, 1 × protease inhibitor cocktail) and sonicated to an average size of 500-bp. Fragments were diluted, blunt-end repaired, and ligated with T4 ligase for 24 hours at 4. Reverse crosslinking was carried out at 65 °C for 20 hours with proteinase K. Protein-free DNA was purified as template for nested PCR using two sets of primers. Primers targeting endogenous *Oct4* enhancer are as follows. 1^st^ set, Forward 1: ACAGGCACTCTGAGGGCTAT, Reverse 1: TCGTTCAGAGCATGGTGTAGG; 2^nd^ set, Forward 2: GTAATGGGATCCTCAGACTGGG, Reverse 2: AGGCTGTGTGATTCACCCTG. Primers targeting transgenic *Oct4* enhancer are the same as those targeting endogenous enhancer, except for the Forward 2: GTAATGGGATCGTGACCCAAGG (Fig. 2). Purified PCR products were further sonicated to an average size of 200-bp, which were sequenced using an Illumina HiiSeq2000 Sequencer.

### Computational analysis of sonication-based 4C libraries

4C libraries were sequenced using 90-bp paired-end reads. Using this method, we extracted 20-bp end tags from forward and reverse reads and aligned them to the mouse reference genome assembly (mm10) separately using Burrows-Wheeler Aligner (BWA^45^). We retained only uniquely mapped reads, with both paired end reads of mapping quality MAPQ >20. Junction reads were identified as one end-tag mapping uniquely to the “bait” region and the other to genomic locations >300-bp away on the same chromosome or different chromosome. We paid particular attention to distal junction reads in which two tags were either 1) on the same chromosome separated by >10 kb, or 2) on different chromosomes. Distal junction reads were further processed to identify distal chromatin interactions. Tags within a 100-bp window were interpreted as PCR products from a single ligation event and merged as one unique distal interacting site. Unique distal sites with only one read were excluded as background noise.

### Significantly enriched interacting domain calling

We applied a statistical model to identify contact regions with significantly higher interaction frequency than that expected from background^10^. Every interacting site *i* on chromosome *W* with length *Lw* was examined within window *w* with length *lw*. The number of interacting sites was defined as *C*_*i,w*_, and a *z* score was assigned to the window based on the following calculation:


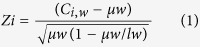


in which *μw* is the expected number of interacting sites in window *w* on chromosome *W*.

We then applied an FDR-based approach to determine statistical significance of each interacting site. We randomly permutated z-score data obtained using the above calculations 100 times for every chromosome, and selected sites with FDR ≤ 0.05 as significant interacting sites. An FDR for each site was calculated by counting randomly permutated z-scores that were larger than the experimentally determined z-score. All interacting sites within +/− 1 Mb range of significant interacting sites were merged into an enriched domain. Overlapping domains, if any, were further merged into a final candidate region.

### Integrative association study

We retrieved ChIP-seq data for histone modifications, DHS data and RNA-seq data from ENCODE Project Portal. RNA-seq raw data were processed using Tophat and Cufflinks. ChIP-seq peak files of 15 DNA-binding proteins were retrieved from the GEO database (GSE11431). A file containing genome-wide 5-hmc peaks in mouse ES cells was provided by Dr. Hao Wu^28^. TTSs and CpG sites were retrieved from UCSC Genome Bioinformatics site. DNA replication timing data for the mouse genome in 3 ES cell lines and 3 iPS cell lines were downloaded from the GEO database. The cell lines and GEO accessions are as follows: 46C (GSM450272), D3 (GSM450273), TT2 (GSM450274), iPSC (GSM450275), iPSC 1D4 (GSM450276), iPSC 2D4 (GSM450277). Segments with mean replication timing ratio above one (log2 transformed early/late replication timing ratio above zero) were defined as early replication regions.

### Other methods

Statistical analysis was carried out using R (http://www.r-project.org/). Conversion of genomic coordinates between genome assemblies was executed using the liftOver tool from the UCSC Genome Bioinformatics Site.

## Additional Information

**How to cite this article**: Cai, M. *et al.* Analysis of a transgenic *Oct4* enhancer reveals high fidelity long-range chromosomal interactions. *Sci. Rep.* **5**, 14558; doi: 10.1038/srep14558 (2015).

## Supplementary Material

Supplementary Information

Supplementary Table 1

Supplementary Table 2

Supplementary Table 3

Supplementary Table 4

## Figures and Tables

**Table 1:** Summary of metrics in 4C-seq analysis.

**cell line**	**BR**	**# read pairs**	**# sites**	**# significant sites**	**# merged regions**	**# overlapping regions**		
MES	1	12351814	3931	953	97	43		
	2	12096428	3131	713	85			
MES-E	1	11682797	1863	424	76	30	13*	9**
	2	11912574	1597	359	66			
MES-G	1	11895568	6983	1487	83	42		
	2	12230450	7734	1808	95			
MIPS-E	1	10136007	5055	1096	83	43	24*	
	2	12134379	4440	1041	93			
MIPS-G	1	11801371	7254	1680	90	47		
	2	11788007	6727	1568	86			

Shown are counts of overlapping regions between biological replicates, together with counts of overlapping regions in analysis of the endogenous and transgenic *Oct4* enhancer. Asterisks (*) indicate counts of regions consistently identified in endogenous and transgenic enhancer interactomes. Asterisk (**) indicates the count of regions consistently identified in MES and MIPS. BR, biological replicates.

**Table 2:** Counts of genes identified around 4C sites.

**cell line**	**BR**	**# genes**	**# overlapping genes**	
MES	1	734	198	
	2	563		
MES-E	1	359	99	21*
	2	301		
MES-G	1	1014	305	
	2	1195		
MIPS-E	1	804	236	63*
	2	755		
MIPS-G	1	1137	384	
	2	1069		

A substantial number of gene interactions overlap in two biological replicates. Asterisk (*) indicates counts of genes consistently identified in both the endogenous and transgenic enhancer interactomes.

**Table 3:** Transcription factor enrichment around 4C sites identified in 10 datasets.

**cell line**	**BR**	**Transcription Factors**				
		**Oct4**	**Klf4**	**Tcfcp2I1**	**Esrrb**	**Zfx**
MES	1	4.507e-05	1.584e-09	1.762e-08	<2.2e-16	/
	2	7.101e-07	8.181e-10	5.045e-12	<2.2e-16	2.452e-09
MES-E	1	4.121e-06	2.997e-06	5.473e-05	3.123e-13	1.474e-06
	2	5.949e-06	7.449e-05	2.517e-05	7.612e-11	0.0001991
MES-G	1	<2.2e-16	<2.2e-16	<2.2e-16	<2.2e-16	<2.2e-16
	2	3.81e-16	<2.2e-16	<2.2e-16	<2.2e-16	<2.2e-16
MIPS-E	1	6.323e-13	<2.2e-16	4.973e-13	<2.2e-16	1.29e-11
	2	3.71e-07	5.96e-14	1.405e-08	<2.2e-16	6.139e-12
MIPS-G	1	<2.2e-16	<2.2e-16	<2.2e-16	<2.2e-16	<2.2e-16
	2	<2.2e-16	<2.2e-16	<2.2e-16	<2.2e-16	<2.2e-16

*p*-values were calculated using the Wilcoxon rank-sum test of differences between tag counts in regions +/− 1 kb of 4C sites and randomly selected genomic sites.
