# Supplementary material for: Analysis of a transgenic Oct4 enhancer reveals high fidelity long-range chromosomal interactions
Source: Sci Rep. 2015 Oct 5;5:14558. doi: 10.1038/srep14558 (PMC4592970; doi:10.1038/srep14558)
Supplement: Supplementary Information [file srep14558-s1.pdf]

Analysis of a transgenic *Oct4* enhancer reveals high fidelity long-range chromosomal interactions

Mingyang Cai, Fan Gao, Peilin Zhang, Woojin An, Jiandang Shi, Kai Wang & Wange Lu

Supplementary Table 1

- Significant sites in 10 datasets (MES-1, MES-2, MES-E-1, MES-E-2, MES-G-1, MES-G-2, MIPS-E-1, MIPS-E-2, MIPS-G-1, MIPS-G-2)

Supplementary Table 2

- Overlapping regions between biological replicates of 5 experimental contexts (MES, MES-E, MES-G, MIPS-E, MIPS-G), between MES-E and MES-G (MES-E & MES-G), between MIPS-E and MIPS-G (MIPS-E & MIPS-G), and between MES and MIPS (MES & MIPS).

Supplementary Table 3

- Overlapping genes between biological replicates of 5 experimental contexts (MES, MES-E, MES-G, MIPS-E, MIPS-G)

Supplementary Table 4

- Overlapping genes between MES-E and MES-G (MES-E & MES-G), and between MIPS-E and MIPS-G (MIPS-E & MIPS-G).

* Contents enclosed with brackets () are sheet names in the excel file.
